# Warning signals only support the first action in a sequence

**DOI:** 10.1186/s41235-023-00484-z

**Published:** 2023-05-12

**Authors:** Niklas Dietze, Lukas Recker, Christian H. Poth

**Affiliations:** Department of Psychology, Neuro-Cognitive Psychology and Center for Cognitive Interaction Technology, Bielefeld University, P.O. box 10 01 31, 33501 Bielefeld, Germany

**Keywords:** Phasic alertness, Action, Cognitive control, Arousal, Pupil, Choice reaction

## Abstract

Acting upon target stimuli from the environment becomes faster when the targets are preceded by a warning (alerting) cue. Accordingly, alerting is often used to support action in safety-critical contexts (e.g., honking to alert others of a traffic situation). Crucially, however, the benefits of alerting for action have been established using laboratory tasks assessing only simple choice reactions. Real-world actions are considerably more complex and mainly consist of sensorimotor sequences of several sub-actions. Therefore, it is still unknown if the benefits of alerting for action transfer from simple choice reactions to such sensorimotor sequences. Here, we investigated how alerting affected performance in a sequential action task derived from the Trail-Making-Test, a well-established neuropsychological test of cognitive action control (Experiment 1). In addition to this task, participants performed a classic alerting paradigm including a simple choice reaction task (Experiment 2). Results showed that alerting sped up responding in both tasks, but in the sequential action task, this benefit was restricted to the first action of a sequence. This was the case, even when multiple actions were performed within a short time (Experiment 3), ruling out that the restriction of alerting to the first action was due to its short-lived nature. Taken together, these findings reveal the existence of an interface between phasic alertness and action control that supports the next action.

## Significance statement

Alerting humans using warning stimuli is assumed to help them handle situations that call for immediate action. We show, however, that the benefits of alerting are much more limited than previously thought. Previous studies investigated alerting effects on actions consisting of a single step action, but in real-life, actions consist of a sequence of steps. We discovered that even though alerting does improve perception and action also in action sequences, these improvements were restricted to only the first action in the sequence. This argues that warning stimuli support behaviour only briefly, for a single action step, which calls into question their ubiquitous application in safety-critical situations.

## Introduction

Human goal-directed behaviour requires sequential actions that work in concert to achieve a given task (Ballard et al., 1995; Hayhoe & Ballard, 2005; Land & Tatler, 2009; Tatler et al., 2005). A supposedly simple task as turning a car, requires a number of actions, such as signalling direction, regulating speed, and turning the steering wheel. In time-sensitive situations as driving, it is vital for one’s safety that actions quickly address the challenges posed by the environment. To support immediate action in such situations, the human brain is equipped with alerting mechanisms assumed to speed up perception and action (Hackley, 2009; Petersen & Posner, 2012). Based on warning stimuli from the environment, these mechanisms elicit a temporary increase of arousal that heightens the readiness for perception and action known as phasic alertness (Posner & Petersen, 1990; Sturm & Willmes, 2001). Even though most goal-directed behaviour relies on sequential actions, the phasic alertness mechanisms and their impact on action have been studied only in highly artificial laboratory choice reaction tasks that only require a simple key press. Therefore, it is unknown how phasic alerting affects sequential actions in more complex tasks, and whether warning stimuli indeed support action in more realistic situations. This is surprising, because in hazard control and safety-critical contexts, warning stimuli are used ubiquitously to improve sequential actions, such as driving (Wogalter & Mayhorn, 2005) and surgical interventions (Cho et al., 2013).

For laboratory tasks requiring a single action, it is well established that phasic alertness affects behaviour substantially: Acting on visual target stimuli is improved when these targets are preceded by visual (Asanowicz & Marzecová, 2017; Dietze & Poth, 2022, in revision; Fan et al., 2002, 2005) or auditory (Dietze & Poth, in revision; Fuentes & Campoy, 2008; Ishigami & Klein, 2010; Poth, 2020; Seibold, 2018) warning stimuli (so-called alerting cues) that induce the state of phasic alertness. Compared with conditions without alerting cues, alerting reduced reaction times in speeded choice tasks (Dietze & Poth, 2022; Fan et al., 2002; Hackley, 2009; Poth, 2020), improved sensitivity in visual discrimination tasks (Kusnir et al., 2011), and made visual processing for object recognition start earlier (Petersen et al., 2017) and proceed faster (Haupt et al., 2018; Matthias et al., 2010; Petersen et al., 2017; Wiegand et al., 2017). In sum, these findings suggest that alerting affects cognitive processing throughout all processing stages, from perceptual encoding (Kusnir et al., 2011; Matthias et al., 2010; Petersen et al., 2017), over response selection (Hackley & Valle-Inclán, 1998, 2003; Posner, 1978), up until response execution (Posner, 1978; Posner & Petersen, 1990). Phasic alertness seems to support fast action, even though alerting cues offer no information about which responses should be performed (Fan et al., 2002; Hackley, 2009; Poth, 2020). In addition, alerting sometimes improves reaction times at the cost of more erroneous actions (Han & Proctor, 2022a; McCormick et al., 2019; Posner et al., 1973) and modulates cognitive control by increasing reaction time differences between conditions with and without cognitive conflicts (Fischer et al., 2010; Nieuwenhuis & de Kleijn, 2013; Schneider, 2019, 2020; Weinbach & Henik, 2012b). This indicates that phasic alertness does not prioritise specific pieces of information (in contrast to selective attention), but that it supports fast action as a non-specific and process-general state of readiness for perception and action (cf. Bundesen et al., 2015).

Phasic alertness seems to optimise cognitive processing for action, but it is assumed to do so very rapidly and only for relatively short periods after the alerting cues (Matthias et al., 2010; Sturm & Willmes, 2001). If phasic alertness was limited to short time-windows, then it might only affect the next sensorimotor action and should be restricted to relatively simple actions, since complex or difficult actions often require longer reaction times (Henry & Rogers, 1960). However, for simple choice reactions, it has recently been found that both visual and auditory alerting effects on behaviour persist for at least 1000 ms after the cue was presented (Dietze & Poth, in revision). This is also supported by pupil size data as a proxy for arousal: Elevated pupil sizes were found between 500 and 1500 ms after cue onset (Petersen et al., 2017). In this time-window, one might perform one to two actions of a fairly simple task, so that alerting could extend from the first to the second action. However, many perceptual and cognitive processes seem to unfold in episodes (Poth, 2017; Schneider, 2013) that can be linked with simple actions such as eye movements (Poth & Schneider, 2016a, 2016b, 2018; Poth et al., 2015) or action steps of action sequences. Given such an episodic nature of perception and cognition for action control, one might also assume that alerting would be limited to the current processing episode and thus only affect the next single action (Foerster & Schneider, 2013; Foerster et al., 2011, 2012). Therefore, it remains completely unknown if even subsequent actions can be affected by alerting.

Several studies have identified sequential effects of alerting cues with variable cue-target onset asynchronies (CTOAs) or foreperiods (Han & Proctor, 2022b; Los et al., 2001, 2021; Steinborn et al., 2008, 2009, 2010; Van der Lubbe et al., 2004). The interval between the cue and target enables a preparatory state which contributes to the upcoming action (Hackley & Valle-Inclán, 2003). Participants develop temporal expectations causing faster reaction times at longer CTOAs (Niemi & Näätänen, 1981). However, not only the length of the current CTOA is an important determinant for phasic alertness but also the length of the previous CTOA. Reaction times in a trial with a shorter CTOA are hampered when preceded by a trial with a longer CTOA. In contrast, reaction times in a trial with a longer CTOA are not affected by the previous trial (Han & Proctor, 2022b; Los et al., 2001; Steinborn et al., 2008, 2009, 2010; Van der Lubbe et al., 2004). This asymmetric pattern demonstrates that the temporal context of preceding and present events is crucial for phasic alertness, and this should be particularly important for action sequences that contain both earlier and later responses relative to the alerting cues and the targets.

Here, we ask how phasic alerting affects action in tasks requiring action sequences rather than a single action. To answer this question, we introduced phasic alerting into a well-established task to study cognitive action control of sequential sensorimotor actions, the Trail-Making-Test (TMT) (Crowe, 1998; Reitan, 1971; Salthouse, 2011). In this test, participants have to connect numbered visual targets in a specific sequence, either using pen and paper, or by clicking using computer mouse and monitor (Bowie & Harvey, 2006). A number of measures can then be derived to assess cognitive processes underlying sequential actions, such as visual search, scanning, mental flexibility, cognitive (executive) control, and motor speed (Salthouse, 2011). Thus, in a single test, the TMT provides a broad screening of visual-cognitive action control, which is why it is widely used to assess cognitive impairments in neurological disease (Tombaugh, 2004). Due to the wide range of cognitive processes measured by the TMT, it is also supposed to be a marker for general fluid intelligence and cognitive functionality in everyday life (Salthouse, 2011). With the sequential actions and the diverse cognitive processes required to solve the TMT, the test can be taken as an indicator of more realistic goal-driven behaviour. Nevertheless, as the TMT can be adjusted and applied to laboratory settings (cf. Foerster & Schneider, 2015; Recker et al., 2022), it does provide the experimental control and methodical rigor that is necessary to study the effects of phasic alertness on individual actions.

The goal of the present study was to find out whether and how sequences of goal-directed actions in the TMT were supported by phasic alerting in a similar fashion as single actions in the widely-used choice reaction task. To this end, participants performed a TMT-task (Experiment 1) whose display of all target stimuli was either preceded by an auditory alerting cue (alert condition) or not preceded by such a cue (no cue condition). In addition, the same participants performed a classic choice reaction task (Experiment 2) whose target stimuli were preceded (alert condition) or not preceded (no cue condition) by alerting cues. As a follow-up, additional participants performed a modified and easier version of the TMT-task (Experiment 3) allowing for faster sequential actions. If phasic alerting not only affects single actions but also sequential actions, then we should not only observe alerting effects in the choice reaction task (Experiment 2) but also in the TMT-tasks (Experiments 1 and 3). As described above, phasic alerting effects are assumed to be relatively short-lasting (Matthias et al., 2010; Sturm & Willmes, 2001), so one might assume that the effects are stronger for the first action(s) of a sequence and decline towards the end. However, as argued above, alerting could also be limited to support only the next single action, so that no phasic alerting effects occur after the first response irrespective of the general short-lived nature of alerting effects. In addition to these open questions, we also asked whether phasic alerting effects reflected a characteristic of individual persons, for example, an individual’s capability for briefly increasing their general readiness for perception and action after an alerting cue. In this case, individual participants’ alerting effects in the TMT-task and the choice reaction task should be related, so that participants with large alerting effects in one task should also have large alerting effects in the other task.

## Open practices statement

Experiments 1A and 2A provide our original results for the two experiments, and Experiments 1B and 2B provide a preregistered (https://osf.io/z2jd5) replication of the original findings. The preregistration was amended by the follow-up Experiment 3. All data, analysis code and experiment code (https://osf.io/qne68/) are available on the Open Science Framework.

## Method

### Participants

The same 17 participants performed Experiment 1A and 2A. They were between 19 and 41 years old (*median* = 25 years, 13 females, 4 males). Additional 17 participants performed the replications Experiment 1B and 2B. They were between 18 and 30 years old (*median* = 23 years, 13 females, 4 males). Experiment 3 was also conducted by 17 participants. They were between 20 and 29 years old (*median* = 24 years, 13 females, 4 males). All participants gave written informed consent and reported normal or corrected-to-normal vision before participation. It was also checked that the participants could hear the alerting tone. They received either course credits or were reimbursed with a standard participation fee. The experiments followed the ethical guidelines of the German Psychological Association (DGPs) and were approved by Bielefeld University’s ethics committee.

### Apparatus and stimuli

All experiments took place in the same dimly lit room. Participants were seated in front of the preheated display monitor (warm-up specifications as in Poth & Horstmann, 2017) at a viewing distance of 71 cm with their head on a chin-and-forehead rest while their eye-movements were recorded with an Eyelink 1000 (SR Research, Ottawa, ON, Canada) at a sampling rate of 1000 Hz. The CRT monitor (G90fB, ViewSonic, Brea, CA, USA) ran at a refresh rate of 85 Hz and a resolution of 1024 × 768. Stimuli were controlled using the Psychtoolbox3 extension (Brainard, 1997; Kleiner et al., 2007; Pelli, 1997) for MATLAB R2014b (The MathWorks, Natick, MA, USA). Responses were collected with a standard external computer mouse (09RRC7 Optical mouse, Dell, Austin, TX, USA). The Luminance of visual stimuli was measured using an LS-110 luminance meter (Konica Minolta, Osaka, Japan) and the sound level of auditory stimuli was measured using a SLM01 sound level meter (Tacklife, Shenzhen Temie Technology, Shenzhen, China). All stimuli were initially black figures (1.3 cd/m^2^) presented on a grey background (40.2 cd/m^2^). A filled circle with a diameter of 0.28° of visual angle was used as a fixation point for all experiments. In Experiment 1, the alerting stimulus was a sine tone with a frequency of 700 or 900 Hz and sound level of 70 dB(A). The target stimuli were numbers from 1 to 8 surrounded by black, circular frames with a diameter of 0.85°. Once the numbers were clicked in the correct order, they changed from black (1.3 cd/m^2^) to white (94.5 cd/m^2^). The target locations were generated randomly from a grid (11.2° × 11.2°) with a minimum distance of 1.4° between the targets. In Experiment 2, the same alerting stimulus was used as in Experiment 1. The target stimulus was a black square (0.5° × 0.5°) presented at an eccentricity of 6.5° to the left or right of screen centre. In Experiment 3, we also used the same alerting stimulus as in the previous two experiments. In contrast to Experiment 1, the circular frames had a diameter of 1.42° and the target locations were generated randomly from a grid (6.5° × 6.5°) with a minimum distance of 1.56° between the targets.

### Procedure of Experiment 1

**Fig. 1:**
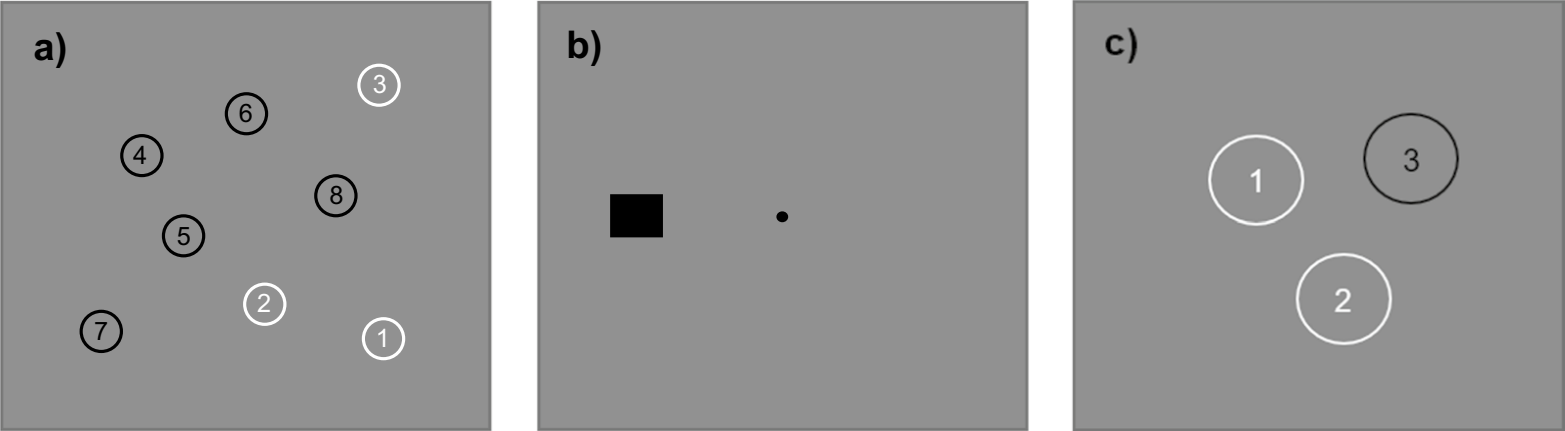
Trial scheme. **a** In Experiment 1, participants responded by clicking on the numbers in increasing order as fast as possible. **b** In Experiment 2, participants responded by pressing the mouse button corresponding to the position of the square as fast as possible. **c** In Experiment 3, participants also responded by clicking on the numbers in increasing order as fast as possible

In Experiment 1, participants conducted a short TMT-task (see Fig. 1a). Here, participants had to respond to the numbers 1 to 8 sequentially with an external computer mouse placed in front of them. At the beginning of the experiment and after 75 trials or 5 broken fixations, a 9-point calibration was conducted. Each trial started with a fixation period of 1000 ms to 4000 ms drawn from a geometric distribution. If participants moved their eyes outside of the fixation window of 2.5° before target onset, the trial was randomly repeated during the remaining sequence. At trial onset, the mouse cursor was positioned to screen centre. In 33% of trials an alerting tone was presented for 50 ms. The CTOAs of 247 ms, 294 ms and 353 ms were also drawn from a geometric distribution with a hazard rate of 0.5 to minimise the anticipatory effects through temporal expectation of the alerting cue (Petersen et al., 2017; Weinbach & Henik, 2012a). After the waiting period, participants were allowed to move their eyes freely and were asked to respond as quickly as possible to the target display, i.e., click the numbered sequence in ascending order. An additional 1000 ms to allow for pupillometry followed after response collection. Each participant conducted 1 practice trial and a total of 210 experimental trials. On average, the time to complete the first experiment was about 60 min.

### Procedure of Experiment 2

In Experiment 2, participants conducted a choice reaction task (see Fig. 1b). The design was identical to Experiment 1 with the exception of the target display. Here, participants only had to respond to the location of one target per trial. Each participant conducted 5 practice trials and again a total of 210 experiment trials. On average, participants time for completion was about 17 min.

### Procedure of Experiment 3

Experiment 3 was the same as Experiment 1 with the exception of the number of targets (see Fig. 1c). Here, participants only had to respond to the numbers 1 to 3 in ascending order. The time for completion was on average about 20 min.

### Statistical analyses

Statistical analyses were performed in R (4.1.2, R Core Team, 2021). For the behavioural analyses, experimental conditions were compared with linear mixed-effect models using the package lme4 (1.1–27.1; Bates et al., 2015), followed up by power simulations using the package simr (1.0.5; Green & MacLeod, 2016). Additionally, pairwise comparisons between no cue and alert conditions were tested with Bayesian *t*-tests using standard settings of the package BayesFactor (0.9.12–4.2; Morey & Rouder, 2021). Practice trials, error trials (Experiment 2: 0.7%) and trials on which participants responded more than 2.5 SD away from the individual mean in each condition (Experiment 1A: 2.3%; Experiment 1B: 1.6%; Experiment 2A: 3.0%; Experiment 2B: 2.8%, Experiment 3: 2.2%) were excluded from the analyses.

For the pupil analyses, raw pupil size measured in arbitrary units was converted to mm. The analyses focused on the period between 500 and 1500 ms after alerting cue onset (cf. Petersen et al., 2017). Prior to the analyses, we computed baseline pupil diameter in the interval 100 ms before cue onset. Then we analysed the changes in pupil size between conditions with the baseline corrected pupil responses. The average difference in pupil size between conditions was computed and compared using pairwise paired *t*-tests with Cohen’s *d*_*z*_ effect size (Cohen, 1988) and Bayesian *t*-tests (Bayes Factor, 0.9.12–4.2; Morey & Rouder, 2021). The same exclusion criteria as the behavioural analyses were applied to the pupil data.

In addition, for both the behavioural and pupil data, Pearson correlation tests were conducted with the mean alerting effect (mean difference between no cue trials and alerting cue trials) for each participant across Experiment 1 and Experiment 2. We also applied a novel method developed by Recker et al. (2022) investigating individual and experimental influences.

## Results and discussion

### Experiment 1

**Fig. 2:**
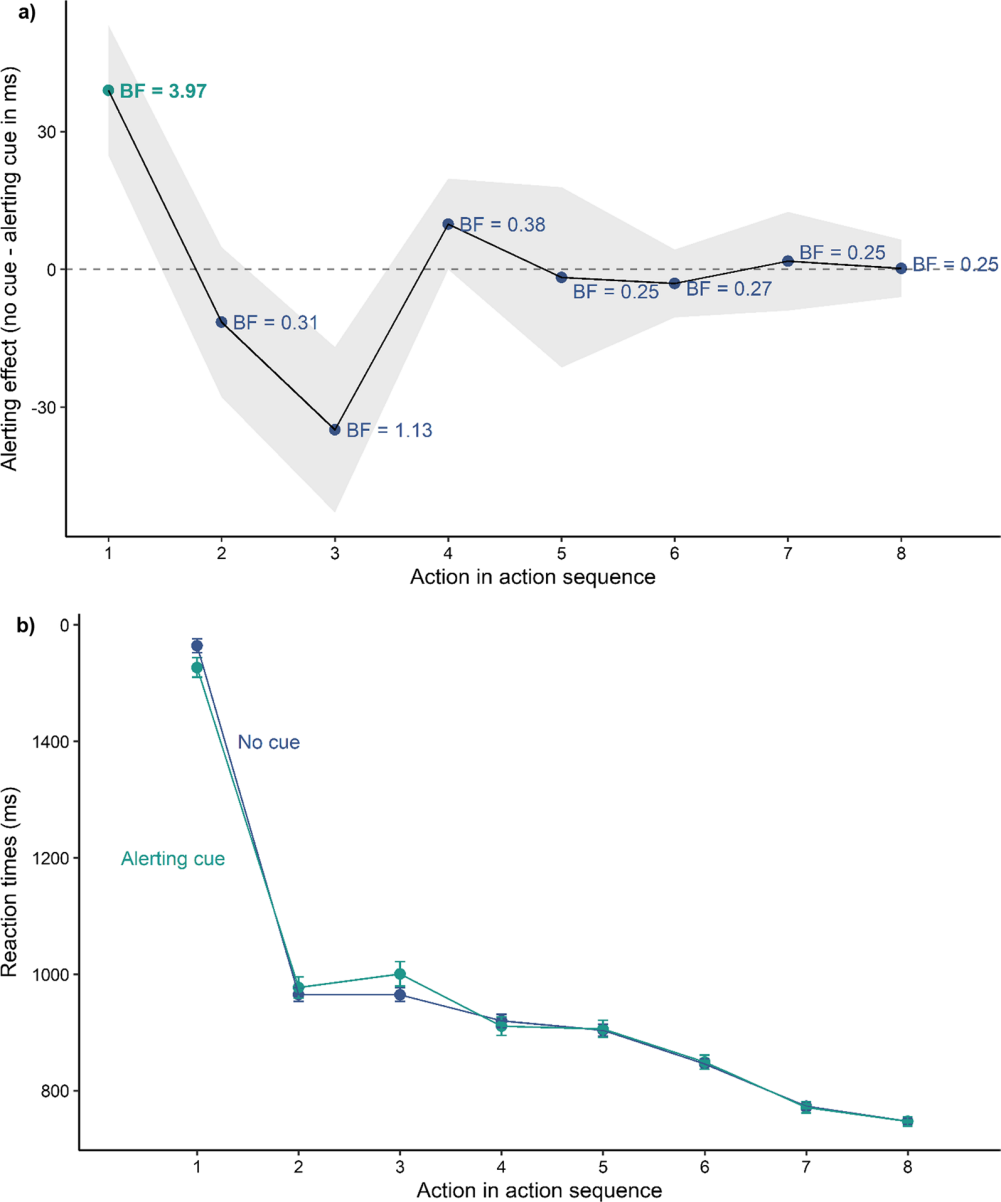
Results of Experiment 1A. **a** Mean alerting effect for all actions. The shaded ribbon represents the standard error of the mean. The Bayes Factors (*BF*_*10*_) have been computed with a Cauchy prior of 0.707 for the mean difference between trials without a cue and with an alerting cue. **b** Mean reaction times for all actions. Error bars represent the standard error of the mean

**Fig. 3:**
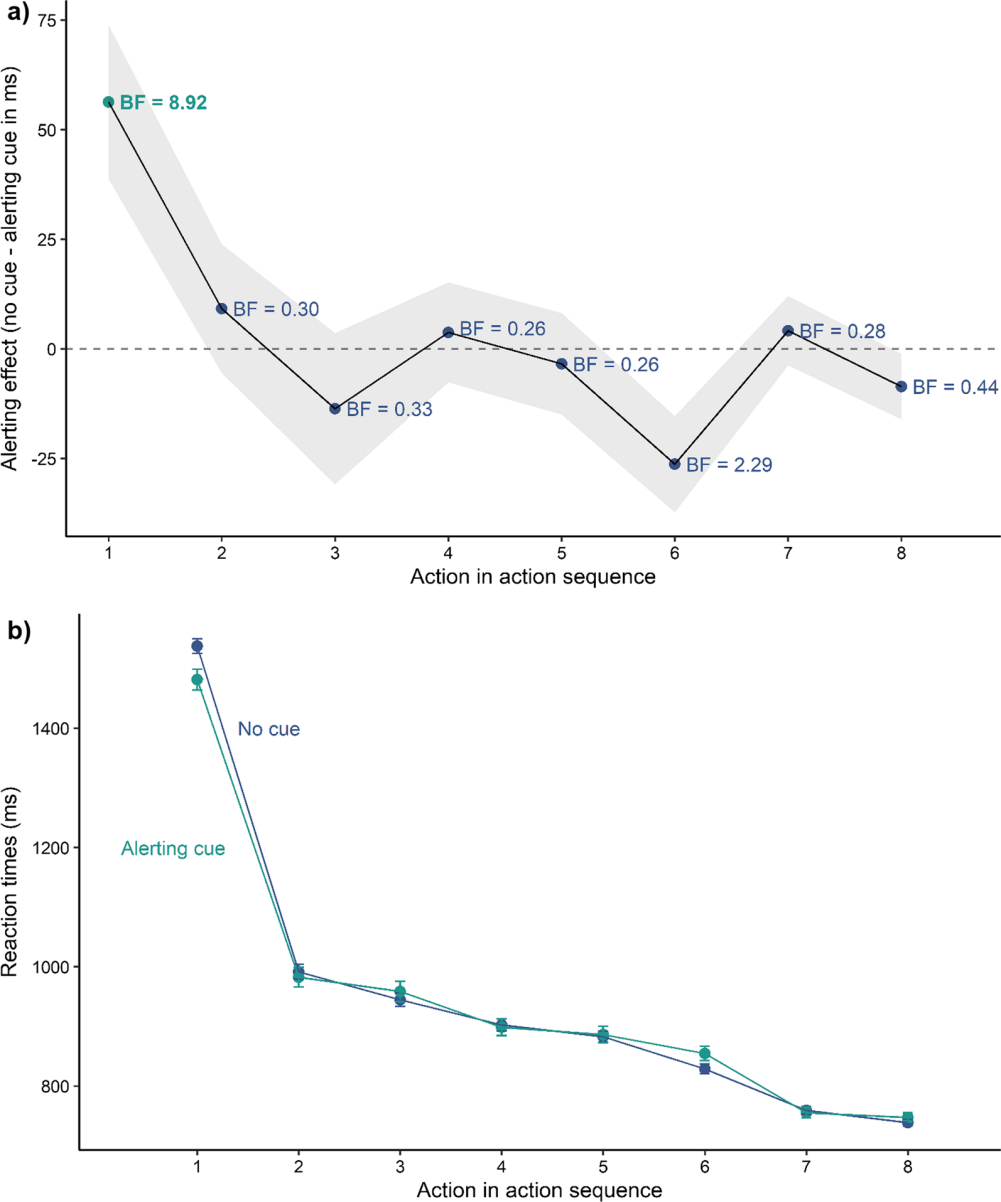
Results of Experiment 1B. **a** Mean alerting effect for all actions. The shaded ribbon represents the standard error of the mean. The Bayes Factors (*BF*_*10*_) have been computed with a Cauchy prior of 0.707 for the mean difference between trials without a cue and with an alerting cue. **b** Mean reaction times for all actions. Error bars represent the standard error of the mean

**Fig. 4:**
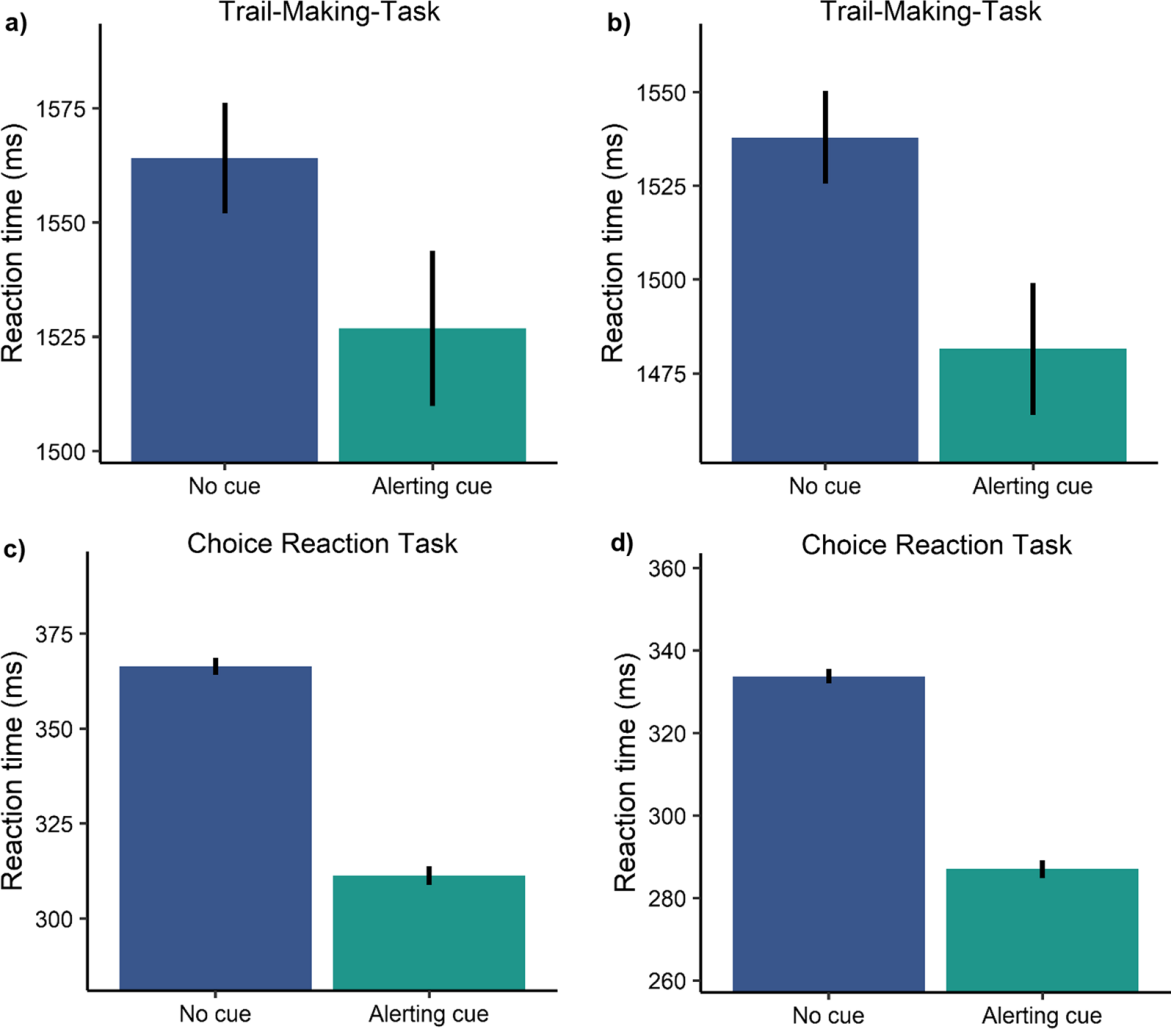
Mean reaction times to the first target of **a** Experiment 1A and **b** Experiment 1B and mean reaction times of **c** Experiment 2A and **d** Experiment 2B. Error bars represent the standard error of the mean

The performance on the TMT-task was analysed with separate linear mixed-effect models including a dummy-coded predictor variable alert (reference = no cue), Euclidean distance to the target as a covariate and a random intercept by participant using the total completion time as well as reaction times to the individual targets as dependent variables to see whether an alerting effect exists across the sequential actions on the target stimuli. We found that only actions on the first target benefited from an alerting cue (see Figs. 2 and 3). In Experiment 1A, we found a strong main effect of alerting (*β* = − 36.77,* t* = − 2.62, *p* < 0.001) showing faster reaction times for trials with a preceding alerting cue than for trials without a cue and a main effect of distance (*β* = 3.14,* t* = 20.04, *p* < 0.001) showing faster reaction times to targets closer to screen centre (see Table 1). On average participants needed 1527 ms (SD = 456 ms) to respond to the first target with a preceding alerting cue and 1564 ms (SD = 459 ms) without a cue (see Fig. 4a). Follow-up power simulations given an effect size of 40 ms resulted in a power of 80.70% [78.11, 83.10]. Experiment 1B replicated these findings by again resulting in a main effect of alerting (*β* = − 49.56,* t* = − 3.45, *p* =  < 0.001) and a main effect of distance (*β* = 3.18,* t* = 19.71, *p* < 0.001) (see Table 1). Participants needed on average 1481 ms (SD = 448 ms) with an alert and 1537 ms (SD = 455 ms) without an alert (see Fig. 4b). The power simulation given the same effect size of 40 ms resulted in a power of 80.10% [77.49, 82.53]. Overall, the present results reveal that alerting had a short-lived effect in the TMT-task. Actions on the first target were facilitated but the alerting effect did not carry-over to the remaining targets.

**Table 1:** Results of the linear mixed-effect models

Experiment	Fixed effects			Random effects	
	(Intercept)	Alert	Distance	*N*_participant_	ICC
1A	986.50** [868.66 to 1104.35]	− 36.77* [− 64.30 to − 9.24]	3.14** [2.83 to 3.45]	17	0.21
1B	951.63** [851.21 to 1052.06]	− 49.56** [− 77.75 to − 21.37]	3.18** [2.86 to 3.49]	17	0.14
2A	366.66** [341.30 to 392.01]	− 55.13** [− 60.22 to − 50.04]	–	17	0.32
2B	334.29** [314.38 to 354.21]	− 47.41** [− 51.38 to − 43.44]	–	17	0.32
3	492.38** [444.36 to 540.41]	− 27.21** [− 36.73 to − 17.68]	2.94** [2.80 to 3.08]	17	0.31

ICC = intraclass correlation; Numbers in squared brackets represent 95% confidence intervals; *p* values are indicated by

^*^ < 0.01,

^**^ < 0.001

In both, Experiment 1A and 1B, the reaction times for the first action (Experiment 1A: *M* = 1552, SD = 458 ms; Experiment 1B: *M* = 1519, SD = 453) were longer than for the subsequent actions (see Figs. 2b and 3b). This is a standard finding for the TMT (Allen et al., 2011), and the longer reaction time for the first action has been assumed to be due to visual and cognitive processes that can be spared for the subsequent actions, such as the initial analysis of the scene (Foerster & Schneider, 2015).

**Fig. 5:**
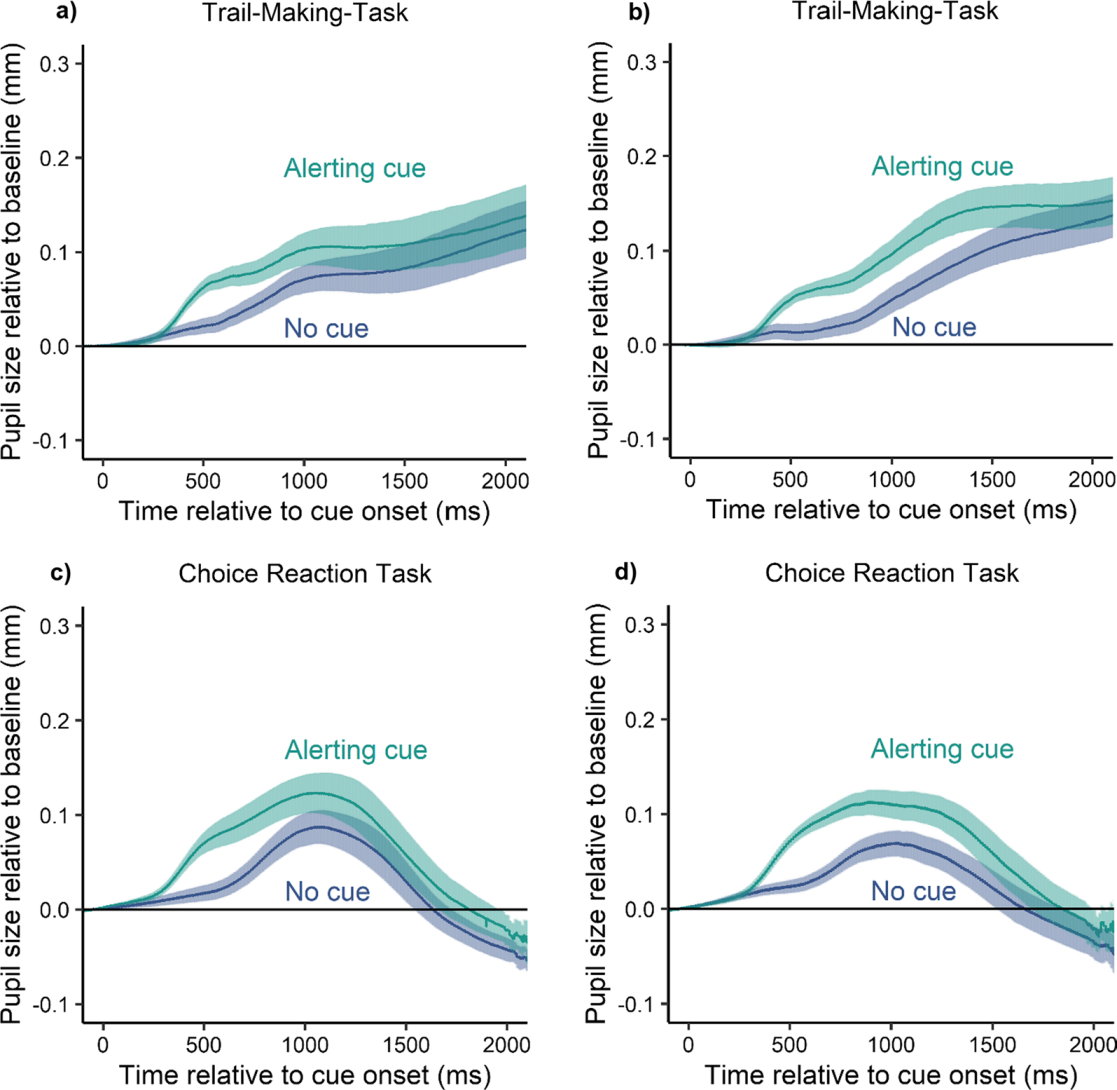
Pupil responses relative to cue onset for **a** Experiment 1A, **b** Experiment 1B, **c** Experiment 2A and **d** Experiment 2B. Lines represent the mean and the shaded ribbon represents the standard error of the mean

For the pupil responses, we found a significant increase in pupil size in the alerting cue condition (*M* = 0.093 mm, SD = 0.064 mm) compared with the no cue condition (*M* = 0.060 mm, SD = 0.052 mm), *t*(16) = 5.10, *p* < 0.001, *d*_*z*_ = 1.24, *BF*_*10*_ = 267 (see Fig. 5a). On average participants’ pupil size was dilated by 0.033 mm when the action sequence was preceded by an alerting cue. In Experiment 1B, we also found a significant increase in the alerting cue condition (*M* = 0.098 mm, SD = 0.056 mm) compared with the no cue condition (*M* = 0.050 mm, SD = 0.049 mm), *t*(16) = 6.38, *p* < 0.001, *d*_*z*_ = 1.55, *BF*_*10*_ = 2396 (see Fig. 5b). Here, participants’ pupil sizes were increased by an average of 0.048 mm. However, differences were only visible in the tested period between 500 and 1500 ms. The average pupil started to converge to roughly the same levels after the initial action.

### Experiment 2

We ran the same analyses as in Experiment 1. In Experiment 2A, alerting significantly reduced reaction times (*β* = − 55.13,* t* = − 21.25, *p* < 0.001) for alert trials (*M* = 311 ms, SD = 73 ms) compared with no cue trials (*M* = 366 ms, SD = 92 ms) (see Fig. 4c and Table 1). Power simulations revealed that an alerting effect size of 40 ms would be detectable with 100% [99.63, 100]. In Experiment 2B, we found similar results (*β* = − 47.41,* t* = − 23.43, *p* < 0.001) showing faster reaction times for alert trials (*M* = 287 ms, SD = 59 ms) than for no cue trials (*M* = 334 ms, SD = 71 ms) (see Fig. 4d and Table 1). The follow-up power simulations also revealed that an alerting effect size of 40 ms would be detectable with 100% [99.63, 100]. As in Experiment 1, average reaction times were around 50 ms shorter with a preceding alerting cue. However, here all participants benefited from the alerting cue, ultimately resulting in a much smaller variance.

For the pupil responses in Experiment 2A we also found a significant increase in pupil size in the alerting cue condition (*M* = 0.059 mm, SD = 0.064 mm) compared with the no cue condition (*M* = 0.101 mm, SD = 0.082 mm), *t*(16) = 5.05, *p* < 0.001, *d*_*z*_ = 1.23, BF_*10*_ = 244 (see Fig. 5c). On average, participants’ pupil size was dilated by 0.042 mm. This was replicated by Experiment 2B. Pupil size in the alerting cue condition (*M* = 0.097 mm, SD = 0.051 mm) was significantly greater than the no cue condition (*M* = 0.049 mm, SD = 0.045 mm), *t*(16) = 7.23, *p* < 0.001, *d*_*z*_ = 1.75, BF_*10*_ = 9416 (see Fig. 5d). Here, participants’ pupil sizes were on average 0.048 mm larger.

### Experiment 3

**Fig. 6:**
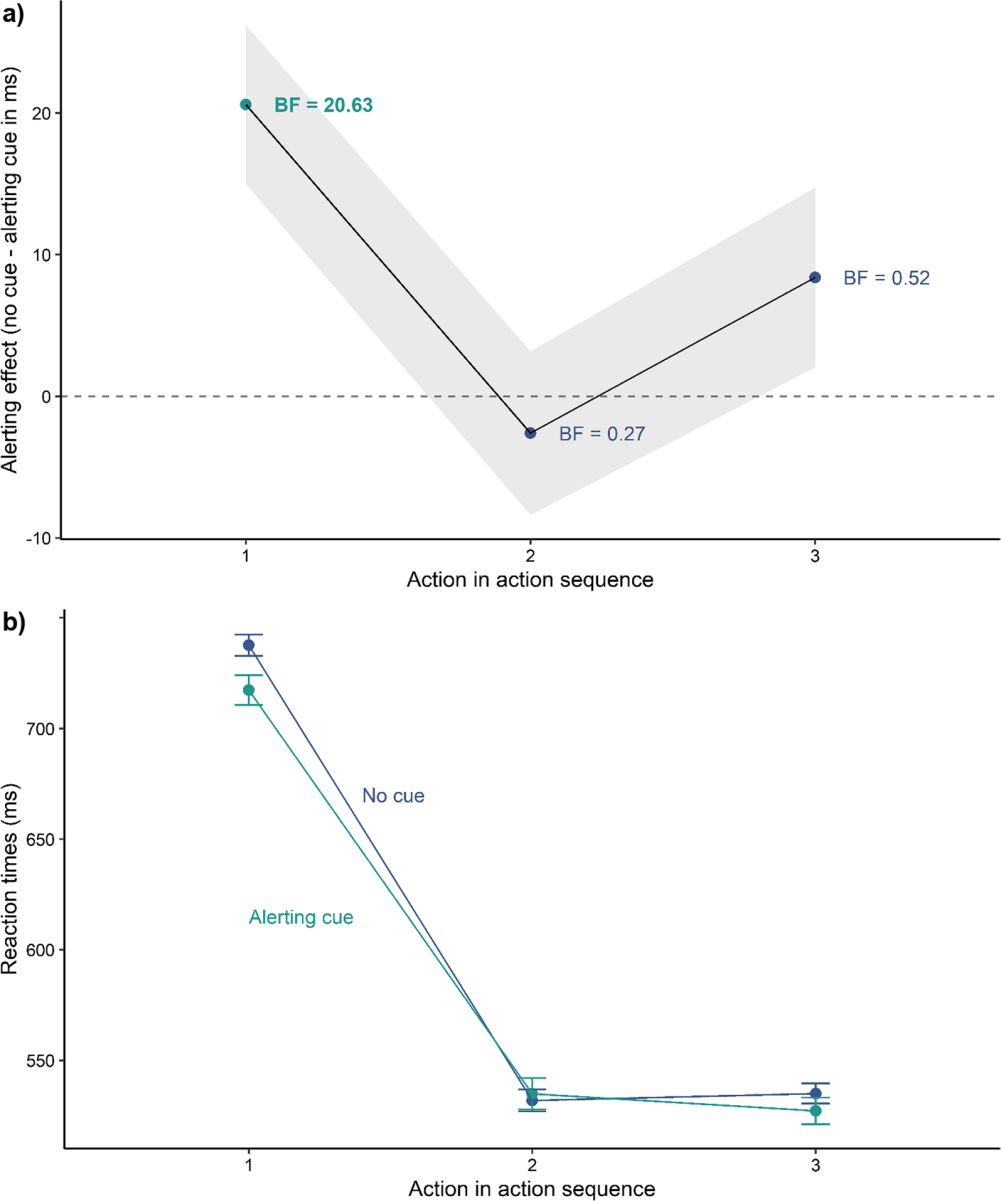
Results of Experiment 3. **a** Mean alerting effect for all actions. The shaded ribbon represents the standard error of the mean. The Bayes Factors (*BF*_*10*_) have been computed with a Cauchy prior of 0.707 for the mean difference between trials without a cue and with an alerting cue. **b** Mean reaction times for all actions. Error bars represent the standard error of the mean

As in Experiment 1, the alerting benefits were restricted to actions on the first target (see Fig. 6) in Experiment 3. We found significantly shorter reaction times for alert trials compared with no cue trials (*β* = − 27.21,* t* = − 5.60, *p* < 0.001) and shorter reaction times to targets closer to screen centre (*β* = 2.94,* t* = 40.74, *p* < 0.001) (see Table 1). On average participants needed 717 ms (SD = 186 ms) to respond to the first target with a preceding alerting cue and 738 ms (SD = 188 ms) without a cue. The follow-up power simulations revealed that an alerting effect size of 40 ms would be detectable with 100% [99.63, 100].

### Correlation between Experiment 1 and Experiment 2

**Fig. 7:**
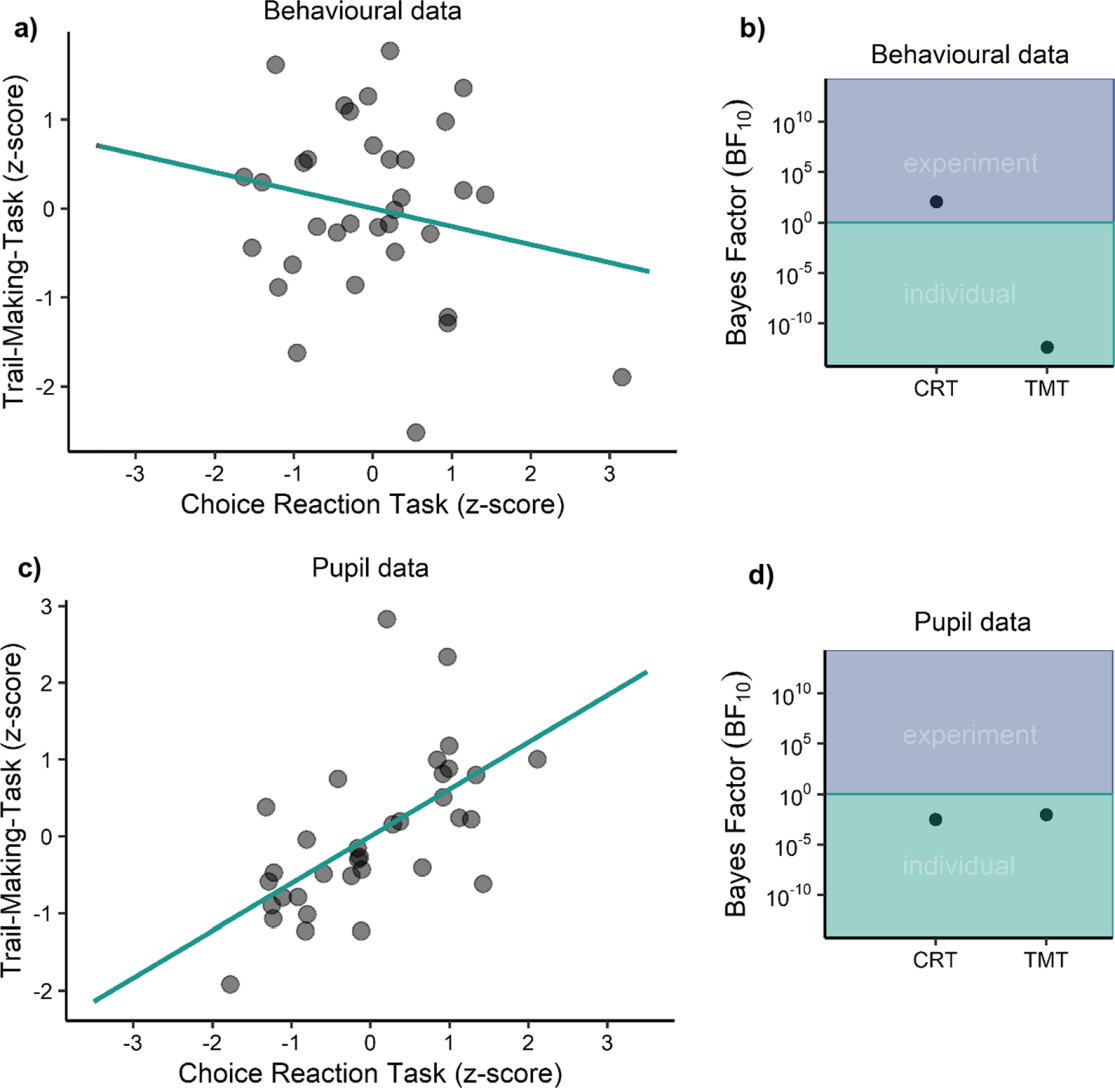
Standardised mean alerting effect (no cue—auditory cue) of each participant for **a** reaction time and **c** pupil size. Bayes Factor (*BF*_*10*_) comparison of fixed versus random effects for **b** reaction time and **d** pupil size

To assess the relationship between both experiments, we collapsed the original results and the replication and computed the mean alerting effect (mean difference between no cue trials and alerting cue trials) for each participant and experiment for reaction times and pupil size respectively. The averages were then submitted to a Pearson correlation test. For the behavioural data, we found that the alerting effects did not correlate with one another (*r*(32) = − 0.203,* p* = 0.250) (see Fig. 7a). In contrast, for the pupil data, we found a significant correlation between the TMT-task and the choice reaction task (*r*(32) = 0.613,* p* < 0.001) (see Fig. 7b). However, well-established experimental effects often suffer from a variance restriction between individuals when put into settings investigating individual differences (Hedge et al., 2018). To corroborate the correlational analyses, we tested whether the alerting effects were experimentally or individually dominated within the respective experiments and dependent measures following a procedure developed by Recker et al. (2022). That is, we compared the variance in the data explained by the experimental effect with the variance due to individual variability. Therefore, we divided the Bayes Factor of a model comprising only interindividual variability (i.e., random intercepts only) by the Bayes Factor comprising only the experimental effects across all participants (i.e., fixed effects only). Results revealed a predominant influence of experimental variability in the reaction times of the choice reaction task (Experiment 2A and B), *BF*_*10*_ = 110. In contrast, the remaining comparisons were all dominated by individual effects, *BF*_*10*_ < 6.14 × 10^–5^ (see Fig. 7b and d).

## General discussion

The present study investigated whether warning stimuli and phasic alertness support sequential, goal-directed sensorimotor actions akin to previously studied single actions. For this purpose, participants conducted a computerised variant of a TMT-task (Experiment 1), followed by a classic alerting paradigm using a choice reaction task (Experiment 2). We discovered that reaction times in both tasks were facilitated when target stimuli were preceded by an alerting cue. However, in the TMT-task, the benefits were restricted to the first action of the sequence. This restriction of alerting effects to the first action could have been either due to the general short-lived nature of phasic alertness or due to an interface of phasic alertness and action control that limits alerting benefits to only the next action step. To distinguish between these explanations, we conducted an additional Experiment 3 using a modified and easier version of the TMT-task that enabled participants to perform multiple actions within the time-frame of the first action in Experiment 1. Even within this time-frame, alerting effects were still restricted to the first action of the action sequence. Thus, rather than just being limited in time, phasic alertness seems also limited to only the next action, suggesting that it interfaces with the mechanisms controlling sequential action.

Surprisingly, we also found that participants’ individual alerting effects on reaction times in the TMT-task were not related to the individual alerting effects in the choice reaction task. In contrast, the individual arousal responses as indicated by the pupil dilations showed a strong relationship between the two tasks. In the TMT-task (Experiment 1), actions on the first target were facilitated by about 50 ms with a preceding alerting cue. For the remaining targets, differences in reaction times without a cue and with an alerting cue were no longer significant. In the choice reaction task (Experiment 2), reaction times to the single visual target were also around 50 ms shorter when preceded by the alerting cue compared with a baseline condition without a cue (cf. classic findings by Callejas et al., 2005; Fan et al., 2002; Hackley & Valle-Inclán, 1998; Posner & Boies, 1971; Poth, 2020). Although, average reaction time benefits were around 50 ms in both tasks, the present data clearly show that findings from highly artificial laboratory tasks relying on single button presses cannot be transferred to more realistic actions consisting of a sequence of sub-actions. The benefits in the TMT-task (Experiment 1) were restricted to the first sub-action and did not extend to the entire sequence. This was also the case when multiple actions were performed within the time required for the first action in our original TMT-task (Experiment 3). Thus, taken together, these findings reveal that warning stimuli and phasic alertness do not support realistic sensorimotor behaviour per se, but only improve the very next action step. While simple individual actions like a button press can be pre-programmed and executed as soon as the target stimulus had been processed, more complex sequential actions require extra steps of planning action steps and updating the overall action plan (Moeller & Frings, 2019; Rosenbaum et al., 1984). Phasic alertness is assumed to enhance perceptual and cognitive processing by triggering a boost of arousal that optimises processing for the current task (e.g., by engaging the locus-coeruleus norepinephrine system that regulates activity widely spread across the cortex, Aston-Jones & Cohen, 2005). The finding that alerting effects were limited to the first action in a sequence could argue that for this arousal boost to affect an action, it must be in contact with the specific cognitive processes controlling the action (e.g., setting up and maintaining a task-set, Dietze & Poth, 2022; Lin & Lu, 2016; response selection, Hackley, 2009; Hackley & Valle-Inclán, 1998, 2003). These processes would need to be established and active already, as could be the case for the first action, but would “loose contact” with the arousal boost as soon as these processes were re-configured for the next subsequent action. As such, the present findings might imply that phasic alertness happened within discrete cognitive episodes for controlling action (cf. Poth, 2017; Schneider, 2013).

The present findings call into question the ubiquitous application of warning systems in safety-critical situations: If warnings stimuli and phasic alertness only support the very next action, then to ensure benefits for behaviour, one must precisely time the warning stimulus to the actions and sub-actions implicated in the target behaviour. This, however, requires that these actions are known in advance and that precise information about their timing is available, which is almost never the case in complex everyday settings (e.g., Benthorn & Frantzich, 1999; Brown et al., 2000). Thus, this argues that warning stimuli should only be used in highly stereotypical situations in which specific actions from the target behaviour can be predicted with high precision and warning stimuli can be timed accordingly.

Surprisingly, we also discovered that individual alerting effects on the first action of the TMT-task (Experiment 1) did not correlate with the individual alerting effects of the choice reaction task (Experiment 2). In other words, whether a given person benefitted from the warning stimulus in the one task did not predict whether the person would also benefit from the warning stimulus in the other task. Thus, for individual persons, the benefits of warning stimuli and phasic alertness seem task-specific or situation-specific. This could indicate that alerting effects on behaviour do not reflect task-general traits in individuals, which would seem at odds with previous studies proposing a trait-like character of phasic alerting effects on behaviour (Aminihajibashi et al., 2020; Aston-Jones & Cohen, 2005; Haupt et al., 2019; Petersen & Posner, 2012). One of these studies found that higher alerting effects on visual processing speed were associated with lower levels of intrinsic functional connectivity within the cingulo-opercular network (Haupt et al., 2019). These studies suggest that there are meaningful individual differences between individual alerting effects that do reflect a basic characteristic of the neuro-cognitive system which could be thought of as a trait. However, that individual alerting effects did not generalise across our two tasks could hint at that the cognitive functions called for by the current task influenced whether or not alerting effects became manifest for a person. In this case, warning stimuli would not only have to be timed with regard to the next action, but the task would also have to be adapted or chosen with respect to the individual person to effectively support behaviour. We observed that alerting effects in the simple choice reaction task (Experiment 2) were dominated by the experimental manipulation, that is the alerting cue condition compared with the no cue condition, whereas alerting effects in the more complex TMT-task (Experiment 1) were dominated by variation between individuals (cf. Recker et al., 2022). The experimental dominance in the choice task indicates that participants’ alerting effects were highly similar and stereotypical, which should have cut the available variance between individuals, and in this way reduced any correlation between the alerting effects in the two tasks. These findings can inform the choice of task for which warning stimuli should be used: For applied situations, non-specific warning stimuli like sirens or alarms work best for simple, highly stereotypical tasks in which humans know what stimuli were going to occur and how they should act upon them (see the discussion above). However, this may be the case only for situations in which the required action follows shortly after the warning stimulus (cf. Langner et al., 2018). For diagnostic purposes, tasks with more complexity may be more informative, as they offer a higher variability between individuals that can be used to test correlations with other person characteristics (cf. Recker et al., 2022). It should be noted, however, that even though we found a strong correlation between the two tasks on the pupil, our sample size (*n* = 34 for this analysis) may have been too small to detect a weaker relationship between the tasks in terms of the behavioural alerting effects. Therefore, dedicated studies are needed to further investigate the extent to which alerting effects generalise across different tasks.

Numerous neuropsychological tests have used alerting effects for behavioural diagnostics of mental disorders such as Alzheimer’s disease and mild cognitive impairment (Festa-Martino et al., 2004; Karpouzian-Rogers et al., 2020; Martella et al., 2014; Tales et al., 2011). The diagnostic conclusions are typically based of the reaction time results (mean difference between no cue trials and alerting cue trials), which presupposes that alerting effects are stable in persons and generalise across different situations. However, as argued above, at least for our present findings (and sample size) the reaction time results seem to reflect alerting only in the context of the specific task. A better diagnostic marker seems to be the arousal responses to the alerting cues that are reflected by the pupil dilation. In the present study, participants’ individual alerting effects on reaction times showed no relationship between the two tasks (Experiment 1 and 2), but individual pupil dilations were strongly correlated. This was the case even though arousal responses are influenced by a wide-range of cognitive processes and stimulus characteristics (Einhäuser, 2017; Mathôt, 2018; Strauch et al., 2022), so that one may have assumed a strong influence of the current task on the pupil response. However, that individual pupil responses to alerting were not specific to the current task could suggest that the pupil responses reflect a trait-like person characteristic that generalises from one task to the next. This is also in line with the finding that the pupil responses were not experimentally dominated but instead depended more strongly on the individual person. That is, the experimental manipulation did not force participants’ pupil into consistent and stereotypical responses, so that the individual influence on the pupil still had enough weight to become manifest in the pupil response. Thus, these findings seem to suggest that the arousal response that is indicated by the pupil is more suitable to capture the individual trait underlying phasic alertness.

The present findings also imply that computerised and eye-tracking-based variants of neuropsychological tests are preferred over paper-and-pencil tests, given their ability to incorporate eye-movements and pupil parameters. These additional measurements allow for a more nuanced and complete assessment of cognitive functions (Bueno et al., 2019; Recker et al., 2022; Recker & Poth, in revision). Like many other neuropsychological tests, the TMT relies on reaction times which typically succumbs a high variability in clinical populations (Kessels, 2019). A computerised variant goes beyond the total completion time as a single outcome variable and allows to measure speed and accuracy of responses to each individual target (Recker et al., 2022). This paves the way for including highly-controlled experimental manipulations (in terms of stimuli and timing) such as phasic alerting, which allow to specifically target cognitive functions that are grounded in neuro-cognitive psychological theory (as discussed by Recker et al., 2022).

Interestingly, it is sometimes assumed that paper–pencil tests akin to the classic TMT minimise unfocused periods or mind-wandering across the task, because the experimenter’s start signal provoked a state of heightened alertness (Schumann et al., 2022). Indeed, such an increase in the (tonic) alertness state should persist across multiple test trials and reduce the variability between reaction times (Fortenbaugh et al., 2017). On the one hand, this should support the reliability of the test, and on the other hand it should set a baseline for perceptual and response readiness that phasic alerting effects were only relative to. Thus, this provides an interesting avenue for future studies investigating how one could enhance computerised tests by means of such a socially-induced tonic alertness and how this would affect their modulation by phasic alertness (Poth, in preparation).

In summary, the present findings show for the first time that phasic alertness affects sensorimotor actions in a sequential task. We found improved reaction times with a preceding warning stimulus but the beneficial effects were restricted to the first action of the action sequence, even when we controlled for the time-frame over which alerting effects unfold. As such, these findings uncover that phasic alertness interfaces with the mechanisms controlling action, ensuring that effects are only directed at the very next action. The findings also show that phasic alerting effects from a classic paradigm using a choice reaction task cannot be transferred to more complex actions. Even though alerting facilitated actions in both experiments and individual arousal responses were similar and correlated across tasks, reaction time benefits for the same participants were not related to each other. This indicates that alerting effects on behaviour could be highly task-specific, which questions the use of warning stimuli in a wide range of fields such as safety control and neuropsychological diagnostics.

## Data Availability

The data, analysis code, experiment code (https://osf.io/qne68/) and pre-registration (https://osf.io/z2jd5) for the replication experiment and follow-up experiment are available on the Open Science Framework.
