# Correction: Warning signals only support the first action in a sequence

**DOI:** 10.1186/s41235-024-00544-y

**Published:** 2024-03-18

**Authors:** Niklas Dietze, Lukas Recker, Christian H. Poth

**Affiliations:** Department of Psychology, Neuro-Cognitive Psychology and Center for Cognitive Interaction Technology, Bielefeld University, P.O. box 10 01 31, 33501 Bielefeld, Germany

## Correction: Cognitive Research: Principles and Implications (2023) 8:29 10.1186/s41235-023-00484-z

The original article contains an error in Fig. 2b whereby the top number on the y-axis was partly covered by the background of the label "b", leaving only the ‘0’ instead of the ‘1600’.

